# Identification of disordered profiles of gut microbiota and functional component in stroke and poststroke epilepsy

**DOI:** 10.1002/brb3.3318

**Published:** 2023-11-20

**Authors:** Duncan Wei, Xiaopu Chen, Jing Xu, Yongling Yin, Xiaotang Peng, Shunxian Li, Wenzhen He

**Affiliations:** ^1^ Department of Pharmacy The First Affiliated Hospital of Shantou University Medical College Shantou Guangdong P. R. China; ^2^ Department of Neurology The First Affiliated Hospital of Shantou University Medical College Shantou Guangdong P. R. China; ^3^ Department of Neurology Shantou University Medical College Shantou Guangdong P. R. China

**Keywords:** diagnosis, function, gut microbiota species, poststroke epilepsy, stroke

## Abstract

**Aims:**

It is estimated that 11.5% of patients with stroke (STR) were at risk of suffering poststroke epilepsy (PSE) within 5 years. Gut microbiota is shown to affect health in humans by producing metabolites. The association between dysregulation of gut microbiota and STR/PSE remains unclear. The aim of this study was to identify potential gut microbiota and functional component in STR and PSE, which may provide a theoretical foundation for diagnosis and treatment of STR and PSE.

**Methods:**

The fresh stool samples were collected from 19 healthy controls, 27 STR patients, and 20 PSE patients for 16S rRNA gene sequencing. Analysis of amplicon sequence variant and community diversity was performed, followed by the identification of dominant species, species differences analysis, diagnostic, and functional analysis of species in STR and PSE.

**Results:**

Community diversity was decreased in STR and PSE. Some disordered profiles of gut microbiota in STR and PSE were identified, such as the increase of *Enterococcus* and the decrease of *butyricicoccus* in STR, the increase of *Escherichia Shigella* and *Clostridium innocuum‐group* and the decrease of *Faecalibacterium* in PSE, and the decrease of *Anaerostipes* in both STR and PSE. Moreover, potential diagnostic biomarkers for STR (*butyricicoccus*), PSE (*Faecalibacterium*), STR, and PSE (*NK4A214_group* and *Veillonella*) were identified. Several significantly dysfunctional components were identified, including l‐tryptophan biosynthesis in STR, fatty acid biosynthesis in PSE, and Stress_Tolerant and anaerobic in both STR and PSE.

**Conclusion:**

The disturbed gut microbiota and related dysfunctional components are closely associated with the progression of STR and PSE.

## Graphical abstract

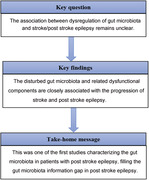

## 1 INTRODUCTION

Cerebral stroke (STR) is an acute cerebrovascular disease, of which ischemic STR accounts for 87% of all STR (Vijayan & Reddy, 2016). Ischemic STR tends to occur in middle‐aged and elderly people, most of whom are over 40 years old. However, with the change of modern lifestyle, the age of ischemic STR is getting earlier and earlier, and many young people have suffered from ischemic STR (Singhal et al., 2013). Poststroke epilepsy (PSE) is defined as epilepsy that occurs once or more after STR without a history of epilepsy on the premise of excluding other brain diseases and metabolic diseases. It is the main cause of acquired epilepsy in adults and most notably in the elderly (Assis et al., 2015; Rodríguez Lucci et al., 2018; Stefan et al., 2014). Research has shown a certain correlation between the lesion site after STR and the occurrence of PSE. Among them, STR involving cortex lesions is the most characteristic risk factor for PSE. The infarction of the superficial STR site, especially in cortical or near‐cortical areas such as frontal cortex, is more prone to develop PSE (Galovic et al., 2021; Zhang et al., 2014). Depending on when the epilepsy begins after the STR, PSE is classified as early onset PSE (EPSE, occurs within 1 week after STR) or late onset PSE (LPSE, occurs 1 week after STR, the peak period is usually 6–12 months after STR) (Xu, 2019). Generally, patients who develop PSE after STR have a poor prognosis and quality of life, and in severe cases, it may even threaten life safety (Arboix et al., 1996; Cleary et al., 2004; Tanaka & Ihara, 2017). Therefore, investigating the potential pathological mechanism of PSE is critical for disease prevention and treatment.

It has been demonstrated that disturbances in the gut microbiome are associated with neurological disorders via the “microbiome‐gut‐brain axis” (Cryan & O'Mahony, 2011). Gut microbiota affects cognitive and behavior functions by producing hormones and metabolites (Mayer et al., 2014; Qamar et al., 2019; Rogers et al., 2016). Some neuroactive molecules produced in the intestine, such as *Pseudomonas* and *Escherichia coli* can synthetize γ‐aminobutyric acid, which is a major inhibitory neurotransmitter in the central nervous system, can cross the blood–brain barrier (Boonstra et al., 2015; Mazzoli & Pessione, 2016). Furthermore, short‐chain fatty acids (SCFAs) produced by gut bacteria contribute to maintaining the integrity of the blood–brain barrier (Burger‐van Paassen et al., 2009). It was speculated that the imbalance of intestinal ecological could be a key factor in the occurrence of epilepsy (Wu et al., 2016). Gómez‐Eguílaz et al. (2018) found that the probiotic treatment could reduce epilepsy frequency and was related to the significant improvement of life quality. However, until now, the role of gut microbiota in PSE remains unknown. Given this, we attempted to identify potential gut microbiota and functional component in STR and PSE compared with healthy individuals. By comparing three groups of different populations, we explored the progression relationship between STR and PSE and explored whether the changes of gut microbiota in PSE patients and STR patients are progressive. The study may be helpful in understanding the microbial change mechanism in STR and PSE, which provide an alternative strategy for diagnosis and treatment.

## 2 METHODS

### 2.1 Study patients

From August 2019 to July 2022, 27 ischemic STR patients and 20 PSE patients diagnosed and treated in the First Affiliated Hospital of Shantou University Medical College, and 19 healthy subjects (CON) from the physical examination center of the same hospital were collected. The general data, including age, gender, smoking status, disturbance of consciousness, aphasia, history of STR, the time of epilepsy onset after STR, and head computed tomography (CT) (or magnetic resonance imaging [MRI]), were collected. Clinical characteristics of studying subjects are shown in Table 1. The Ethics Committee of the First Affiliated Hospital of Shantou University Medical College approved the study protocol, and patients or their legal guardians signed written informed consent.

**TABLE 1:** Clinical characteristic of enrolled individuals.

Group	Gender	Age (year)	Hypertension	Diabetes	Smoking history	Disturbance of consciousness	Aphasia	Limb convulsion	History of stroke	The point in time at which the first seizure occurs after the stroke	Head CT (or MRI)
STR	Female	60	Yes	No	No	No	No	No	First	No	Subacute cerebral infarction
	Female	70	Yes	No	No	No	No	No	First	No	Acute cerebral infarction
	Male	59	Yes	No	Yes	No	No	No	First	No	Acute cerebral infarction
	Female	61	Yes	Yes	No	No	No	No	First	No	Acute cerebral infarction
	Male	58	No	No	No	No	No	No	First	No	Ischemic lesion
	Male	64	Yes	Yes	No	No	No	No	First	No	Acute cerebral infarction
	Female	67	Yes	No	No	No	No	No	First	No	Acute cerebral infarction
	Female	69	Yes	No	No	No	No	No	First	No	Subacute cerebral infarction
	Female	66	Yes	Yes	No	No	No	No	First	No	Infarction
	Male	51	Yes	No	Yes	No	No	No	First	No	Subacute cerebral infarction
	Male	74	Yes	Yes	Yes	No	No	No	First	No	Subacute cerebral infarction
	Male	69	Yes	Yes	No	No	No	No	First	No	Acute cerebral infarction
	Female	54	Yes	No	No	No	No	No	First	No	Acute cerebral infarction
	Male	38	No	No	No	No	No	No	First	No	Acute cerebral infarction
	Female	64	Yes	No	No	No	No	No	First	No	Acute cerebral infarction
	Female	55	Yes	No	No	No	No	No	First	No	Subacute cerebral infarction
	Female	66	Yes	No	No	No	No	Yes	First	No	Subacute cerebral infarction
	Male	62	Yes	Yes	No	No	No	No	First	No	Subacute cerebral infarction
	Female	73	Yes	Yes	No	No	No	Yes	First	No	Subacute cerebral infarction
	Female	61	Yes	Yes	Yes	No	No	No	First	No	Subacute cerebral infarction
	Male	56	Yes	Yes	Yes	No	No	No	First	No	Subacute cerebral infarction
	Male	74	Yes	No	No	No	No	No	First	No	Acute cerebral infarction
	Male	68	Yes	Yes	Yes	No	No	Yes	First	No	Acute cerebral infarction
	Male	45	Yes	No	No	No	No	No	First	No	Subacute cerebral infarction
	Female	74	Yes	No	No	No	No	No	First	No	Acute cerebral infarction
	Male	62	No	No	No	No	No	Yes	First	No	Acute cerebral infarction
	Female	59	Yes	No	No	No	No	No	First	No	Subacute cerebral infarction
CON	Male	64	Yes	No	Yes	No	No	No	No	No	No
	Male	54	Yes	No	Yes	No	No	No	No	No	No
	Female	73	Yes	Yes	No	No	No	No	No	No	No
	Male	71	No	No	No	No	No	No	No	No	No
	Female	57	Yes	No	No	No	No	No	No	No	No
	Female	53	No	Yes	No	No	No	No	No	No	No
	Female	58	Yes	No	No	No	No	No	No	No	No
	Female	62	No	No	No	No	No	No	No	No	No
	Male	51	Yes	No	No	No	No	No	No	No	No
	Male	61	No	No	No	No	No	No	No	No	No
	Female	60	Yes	Yes	No	No	No	No	No	No	No
	Female	56	No	No	No	No	No	No	No	No	No
	Male	51	No	No	Yes	No	No	No	No	No	No
	Male	52	No	No	Yes	No	No	No	No	No	No
	Female	60	No	No	No	No	No	No	No	No	No
	Male	56	No	No	No	No	No	No	No	No	No
	Male	65	No	No	No	No	No	No	No	No	No
	Male	55	No	No	No	No	No	No	No	No	No
	Female	73	Yes	No	No	No	No	No	No	No	No
PSE	Female	67	Yes	Yes	No	Yes	No	No	More	several years later	Ischemic lesion
	Female	72	No	No	No	Yes	No	Yes	One	Within 7 days	Cerebral infarction
	Male	57	Yes	No	Yes	No	Yes	Yes	One	2 week	Massive Cerebral Infarction
	Female	70	Yes	No	No	No	No	Yes	One	That day	Cerebral infarction
	Female	80	No	No	No	No	Yes	No	More	That day	Subacute cerebral infarction
	Male	82	Yes	No	Yes	Yes	Coma	Yes	More	That day	Massive Cerebral Infarction
	Male	52	Yes	Yes	No	Yes	No	Yes	One	1 month after	Lacunar infarct
	Female	62	Yes	No	No	Yes	Coma	Yes	One	6 month after	Cerebral arteriosclerosis
	Female	44	Yes	No	No	No	No	Yes	More	That day	Cerebral arteriosclerosis
	Female	65	Yes	No	No	Yes	Yes	Yes	One	That day	Cerebral arteriosclerosis
	Male	64	Yes	No	No	No	No	Yes	One	After 10 days	Cerebral arteriosclerosis
	Female	51	Yes	No	No	Yes	Coma	Yes	More	16 months later	Ischemic lesion
	Male	85	Yes	Yes	No	No	No	Yes	More	11 months later	Cerebral arteriosclerosis
	Male	65	Yes	No	No	No	No	Yes	More	That day	Cerebral arteriosclerosis
	Male	68	Yes	Yes	No	No	No	Yes	More	After 10 days	Ischemic lesion
	Female	76	Yes	No	No	No	Yes	Yes	One	8 months later	Ischemic lesion
	Male	75	Yes	Yes	Yes	No	No	Yes	One	After 5 days	Ischemic lesion
	Female	82	Yes	No	No	No	No	Yes	More	After 14 days	Lacunar infarct
	Male	80	Yes	No	No	No	Yes	Yes	One	That day	Cerebral arteriosclerosis
	Female	63	Yes	Yes	No	No	No	Yes	More	Within 7 days	Ischemic lesion

Abbreviations: CON, healthy controls; PSE, poststroke epilepsy; STR, stroke.

The inclusion criteria for STR patients were as follows: (1) Patients were diagnosed with ischemic STR by CT or MRI of the brain; (2) patients were 30–90 years; (3) patients were experiences infarction in nonstrategic brain regions (including the subcortex, brain stem, and cerebellum). The exclusion criteria for STR patients were as follows: (1) patients with preexisting dementia history and strategic regional infarction (including hippocampus, thalamus, frontal lobe, cingulate gyrus, angular gyrus, internal capsule, and caudate nucleus); (2) patients took antibiotics or probiotics within 3 months; (3) patients with restrictive diet, gastrointestinal surgery, recent infection, psychosis, severe life‐threatening illnesses, communication deficits, and pregnancy. The inclusion criteria for PSE patients were as follows: (1) Patients had at least two unprovoked (or reflex) seizures occurring more than 24 h apart; (2) patients had epileptic syndrome; (3) epilepsy that occurs after ischemic STR.

### 2.2 Sample collection and processing

Fresh stool samples from 19 CON, 27 STR patients, and 20 PSE patients were obtained within 1 week of admission. Stool samples were collected and immediately transferred to the laboratory for repackaging within 15 min. Subsequently, 200 mg stool samples were put in a 2 mL sterile centrifuge tube and labeled. All specimens were processed within 30 min and stored at −80°C. Stool genomic DNA was extracted as described in the previous study (Li et al., 2008; Shkoporov et al., 2018). A 1% agarose gel was used to evaluate the amount of extracted genomic DNA and stored high‐quality DNA at −20°C. The polymerase chain reaction (PCR) was performed to amplify the V3–V4 region of the bacterial 16S ribosomal RNA gene on the ABI GeneAmp9700. PCR reactions were performed in triplicate, and the products were recovered using AxyPrepDNA Gel Recovery Kit (AXYGEN) Tris_HCl elution and tested by 2% agarose gel electrophoresis.

### 2.3 16S rRNA sequencing and sequencing data processing

The PCR products were quantified using the QuantiFluor‐ST Blue Fluorescence Quantitative System (Promega) at the initial quantitative results of the electrophoresis, and then mixing the corresponding proportions according to the sequencing requirements of each sample. Subsequently, the Illumina MiSeq platform was used for high‐throughput sequencing, and the PE reads obtained from the sequencing were sample separated. The optimized data after quality control were obtained based on the overlap between the double‐end reads. Raw fastq files were de‐multiplexed and quality‐filtered using FLASH Trimmomatic.

### 2.4 Amplicon sequence variant (ASV) analysis

The optimized data were further processed by DADA2 or Deblur to obtain the real sequence information, amplicon sequence variant (ASV). To get the species classification information for each ASV, classify‐sklearn, classify‐consensus‐vsearch, classify‐consensus‐blast, and RDP classifier Bayes algorithm was used to analyze the representative sequences of ASVs and the annotation information of ASVs at different classification levels were obtained. Corresponding abundance information of ASV annotation results in each sample was calculated. Pan/Core species analysis was performed by analysis software of *R*‐3.3.1 (vegan) to describe the changes in total and core species with the increase of sample size. Rank‐abundance curve is one way to analyze microbial diversity, including species abundance and community evenness. The width of the curve in the horizontal direction reflects species richness, whereas the smoothness of the curve reflects the evenness of species in the sample.

### 2.5 Community diversity analysis

Bacterial diversity was measured by α‐diversity and β‐diversity. The α‐diversity is mainly used to study community diversity in a sample by analysis software of mothur‐1.30. A series of α‐diversity indices can be evaluated to obtain information such as richness (sobs, chao, and ace) and diversity (shannon and simpson) of species in environmental communities. The larger the sobs, chao, or ace index, the higher the flora richness. The shannon index and simpson index were used to measure bacterial diversity, which is directly proportional to the shannon index and inversely proportional to the simpson index. The Wilcoxon rank‐sum test was used to compare the α‐diversity indices among the different groups. The β‐diversity was analyzed by using Bray Curtis distances and visualized via principal coordinates analysis (PCoA).

### 2.6 Identification of dominant species and analysis of species differences

The Circos diagram in STR and PSE was obtained to identify the dominant species in gut microbiota species by using R language. According to the obtained community abundance data, Wilcoxon rank‐sum tests were used to test the hypothesis of species between different groups of microbial communities, evaluate the significance level of species abundance differences, and obtain the species with significant differences between groups.

### 2.7 Model prediction analysis

The random forest model was adopted to select the most important diagnostic biomarkers efficiently and quickly through random Forest package in R language. The plotROC package was used to obtain specificity, sensitivity, and the area under a receiver operating characteristic curve (AUC), and the diagnostic ability of all the optimal gut microbiota species was evaluated.

### 2.8 Functional predictive analysis of species

BugBase is a kind of microbial group analysis tools that can be used to predict high level phenotype in the microbiome sample. PICRUSt2 is a software that predicts functional abundance based only on the sequence of marker genes. The function usually refers to KEGG pathway, MetaCyc, Module, and so on. The annotation of KEGG pathway, MetaCyc, and Module was conducted using BLASTP search with an *e*‐value cutoff of 1e − 5.

### 2.9 Statistical analysis

Data analysis and processing were conducted using SPSS 22.0 software. Count data were expressed in terms of the number of cases and percentage (%). The chi‐square test or Fisher exact test was used to compare the differences between groups. Measurement data were expressed as mean ± standard deviation (*x* ± SD). If the measurement data conforms to normal distribution, *t*‐test was used. If the measurement data not conform to normal distribution, nonparametric test was used. *p* < .05 was regarded as statistical significance. The drawings were made using Graphpad 8.0 software.

## 3 RESULTS

### 3.1 Gut microbiota species annotation and evaluation in STR and PSE

**FIGURE 1:**
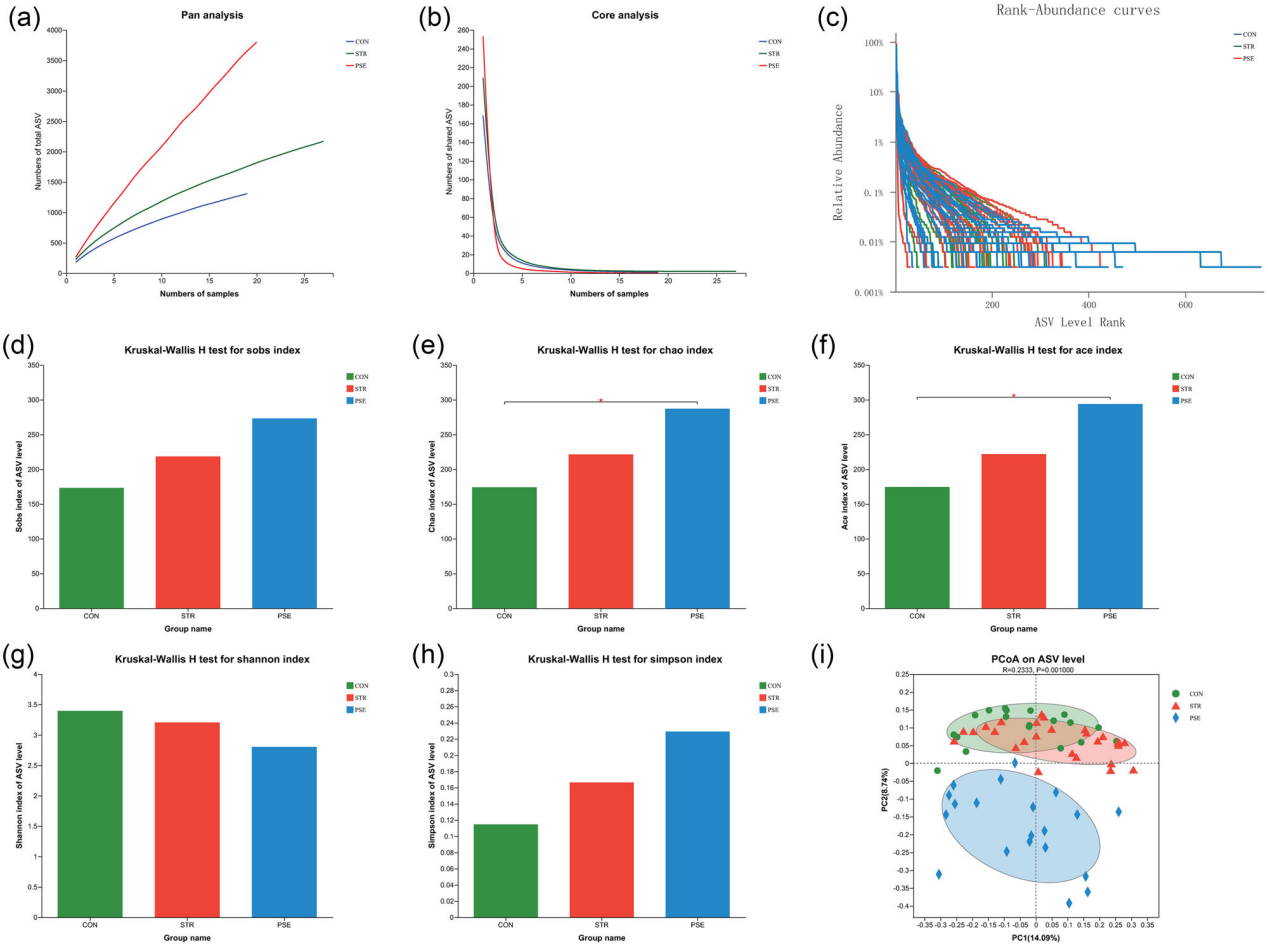
Gut microbiota species annotation and evaluation in stroke (STR) and poststroke epilepsy (PSE): (a) pan analysis; (b) core analysis; (c) rank‐abundance analysis; (d) richness test in sobs index; (e) richness test in chao index; (f) richness test in ace index; (g) diversity test in shannon index; (h) diversity test in simpson index; and (i) principal coordinates analysis (PCoA) analysis. ^*^.01 < *p* < .05.

Pan/core analysis showed that the larger the sample size, the greater the total number of gut microbiota species in STR and PSE (Figure 1a), whereas the smaller the number of core gut microbiota (Figure 1b), and the gut microbiota species in STR and PSE were abundant and evenly distributed (Figure 1c). Compared with CON, the community richness was increased (Figure 1d) in sobs index in STR and PSE. Significantly, community richness was significantly increased in chao and ace indexes in PSE compared with CON (Figure 1e,f). Community diversity was decreased in shannon and simpson indexes in STR and PSE compared with CON (Figure 1g,h). The PCoA of β‐diversity analysis showed a clear separation between CON, STR, and PSE (Figure 1i). It can be seen that there are significant differences in the structure of gut microbiota among CON, STR, and PSE.

### 3.2 Difference analysis of gut microbiota species in STR and PSE

**FIGURE 2:**
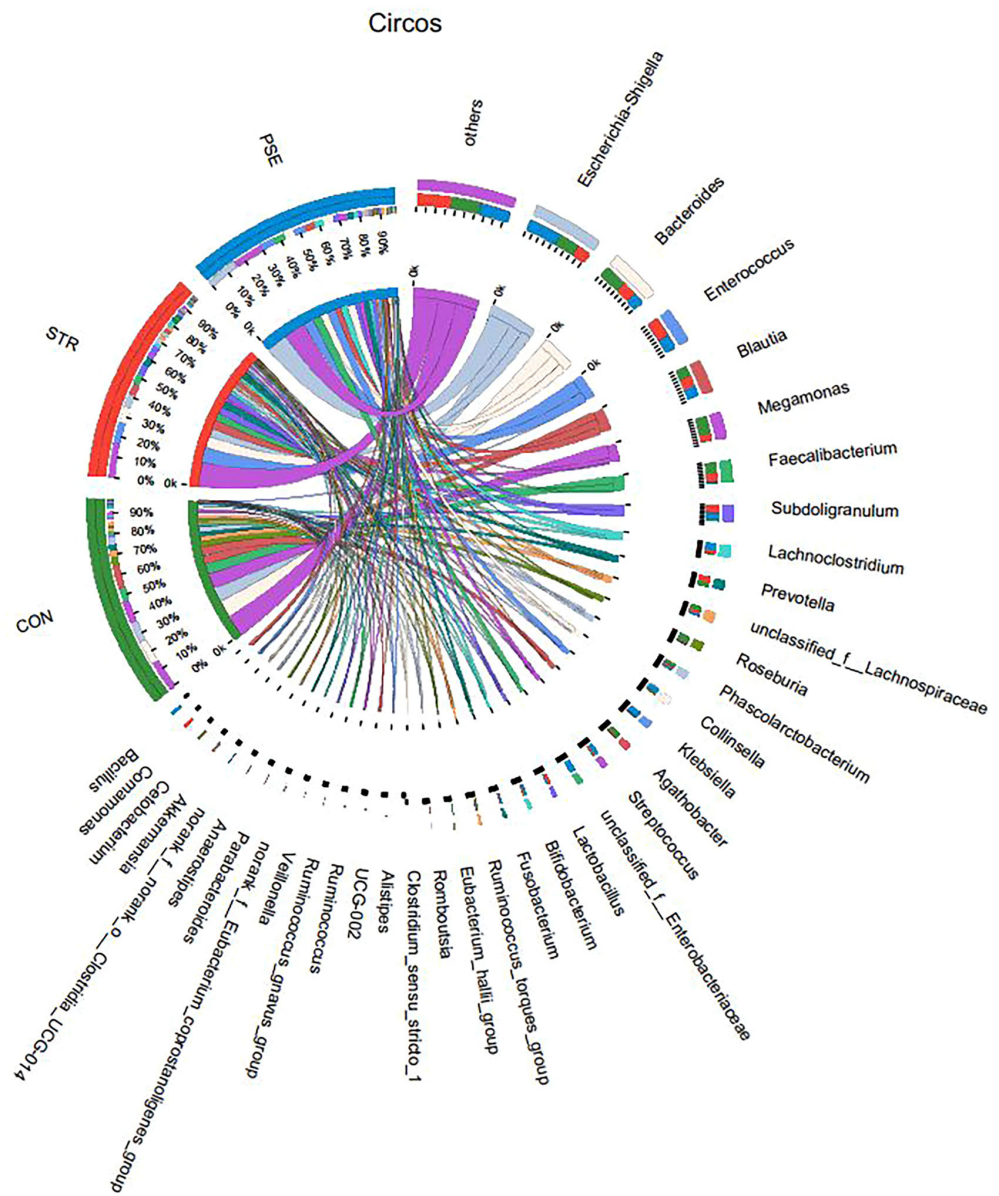
Circos diagram of dominant species in stroke (STR) and poststroke epilepsy (PSE).

**FIGURE 3:**
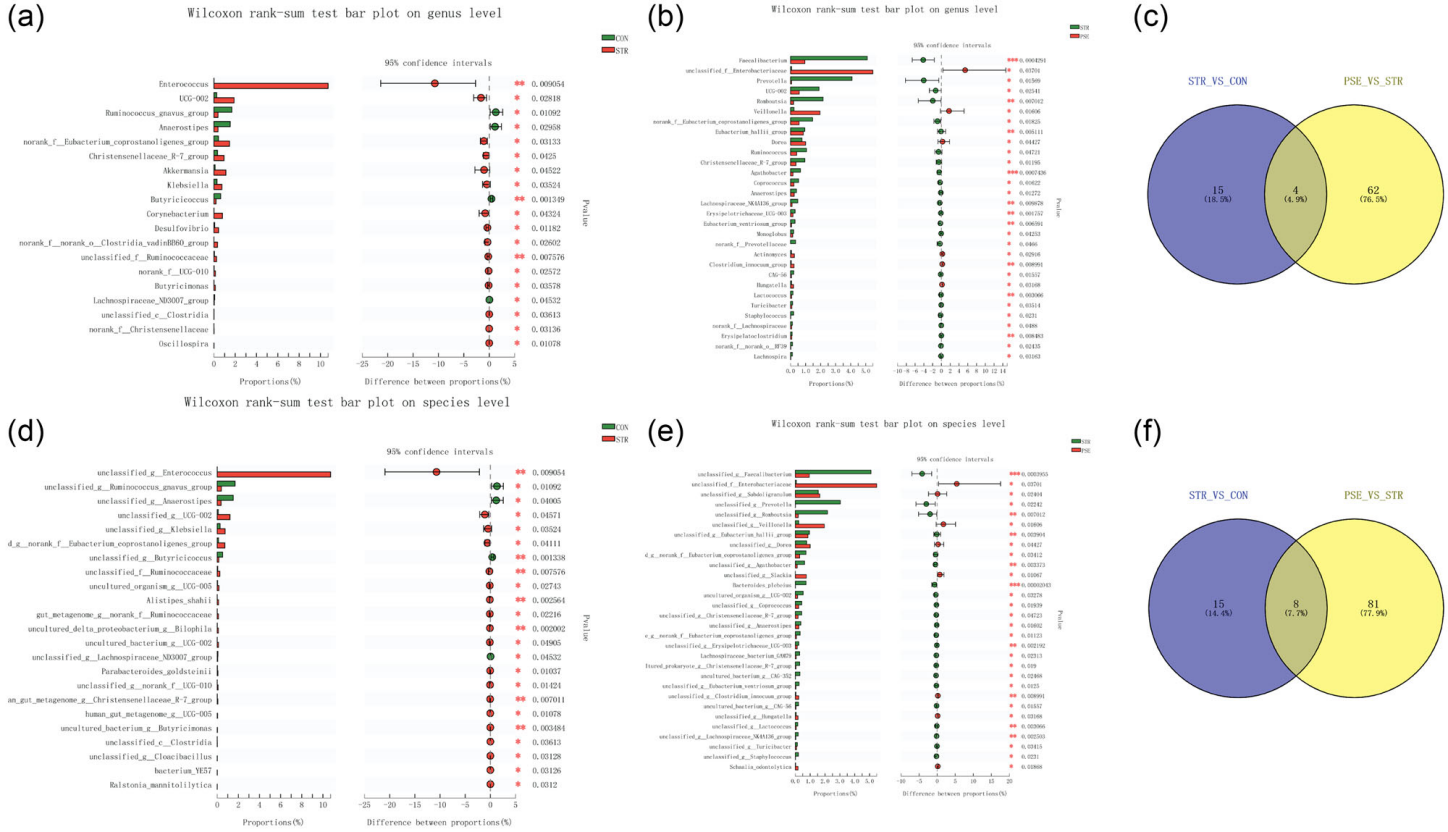
Analysis of differences in gut microbiota between stroke (STR) and poststroke epilepsy (PSE) at the genus/species levels: (a) difference analysis in STR versus CON at genus levels; (b) difference analysis in PSE versus STR at genus levels; (c) common gut microbiota species in STR and PSE at genus levels; (d) difference analysis in STR versus CON at species levels; (e) difference analysis in PSE versus STR at species levels; (f) common gut microbiota species in STR and PSE at species levels.

**FIGURE 4:**
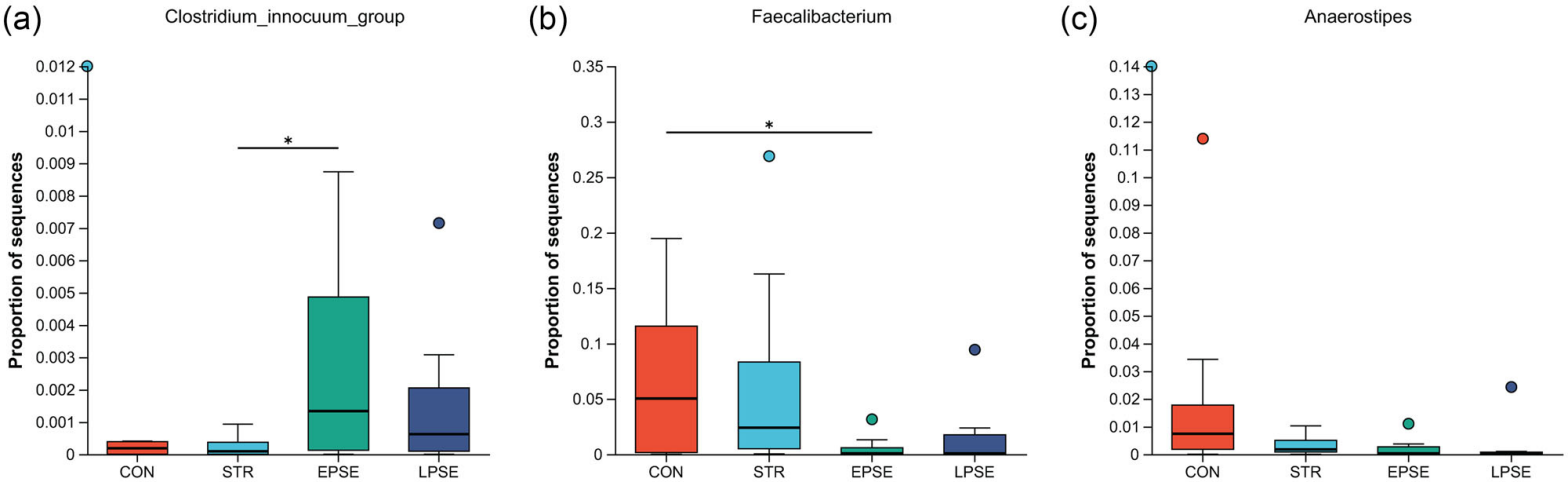
The abundance change of (a) *Clostridium innocuum*‐group, (b) *Faecalibacterium*, and (c) *Anaerostipes* in stroke (STR), EPSE, and LPSE.^*^.01 < *p* < .05.

From the Circos diagram, *Enterococcus* and *Escherichia Shigella* were, respectively, dominant gut microbiota species in STR and PSE at genus level (Figure 2). In order to further identify potential gut microbiota species associated with STR and PSE, difference analysis was performed. At the genus level, the relative abundance of *butyricicoccus* was the most markedly decreased in STR compared with CON (Figure 3a). The relative abundance of *Clostridium innocuum‐group* and *Faecalibacterium* was the most markedly changed in PSE compared with STR (Figure 3b). A total of four common gut microbiota species were identified in both STR and PSE at the genus level (Figure 3c), including *UCG‐002*, *Anaerostipes*, *norank_f__Eubacterium_coprostanoligenes_group*, and *Christensenellaceae_R‐7_group*. At the species level, the relative abundance of *butyricicoccus* was also the most markedly decreased in STR compared with CON (Figure 3d). The relative abundance of *C. innocuum‐group* was also the most significantly increased in PSE compared with STR (Figure 3e). A total of eight common gut microbiota species were identified in both STR and PSE at species levels (Figure 3f), including *unclassified_g__Anaerostipes*, *unclassified_g__norank_f__Eubacterium_coprostanoligenes_group*, *unclassified_g__norank_f__UCG‐010*, *human_gut_metagenome_g__Christensenellaceae_R‐7_group*, *human_gut_metagenome_g__UCG‐005*, *unclassified_g__Cloacibacillus*, *bacterium_YE57*, *and Ralstonia_mannitolilytica*. It is worth nothing that the relative abundance of *Anaerostipes* was markedly decreased in both STR and PSE at the genus level and the species level. In addition, the abundance of *C. innocuum‐group*, *Faecalibacterium*, and *Anaerostipes* was analyzed in the 19 CON, 27 STR patients, EPSE and LPSE of 20 PSE patients (Figure 4). The result showed that the abundance of *C. innocuum‐group* was significantly increased in EPSE compared with STR. Moreover, there was an increased trend of *C. innocuum‐group* in LPSE compared with STR. It is indicated that *C. innocuum‐group* may be significantly associated with EPSE. There was a decreased trend of *Faecalibacterium* in EPSE and LPSE compared with STR. There was a gradual decline of *Anaerostipes* in STR, EPSE, and LPSE. These results further demonstrated the abundance change of *C. innocuum‐group*, *Faecalibacterium*, and *Anaerostipes* in STR and PSE.

### 3.3 Potential diagnostic biomarkers for STR and PSE

**FIGURE 5:**
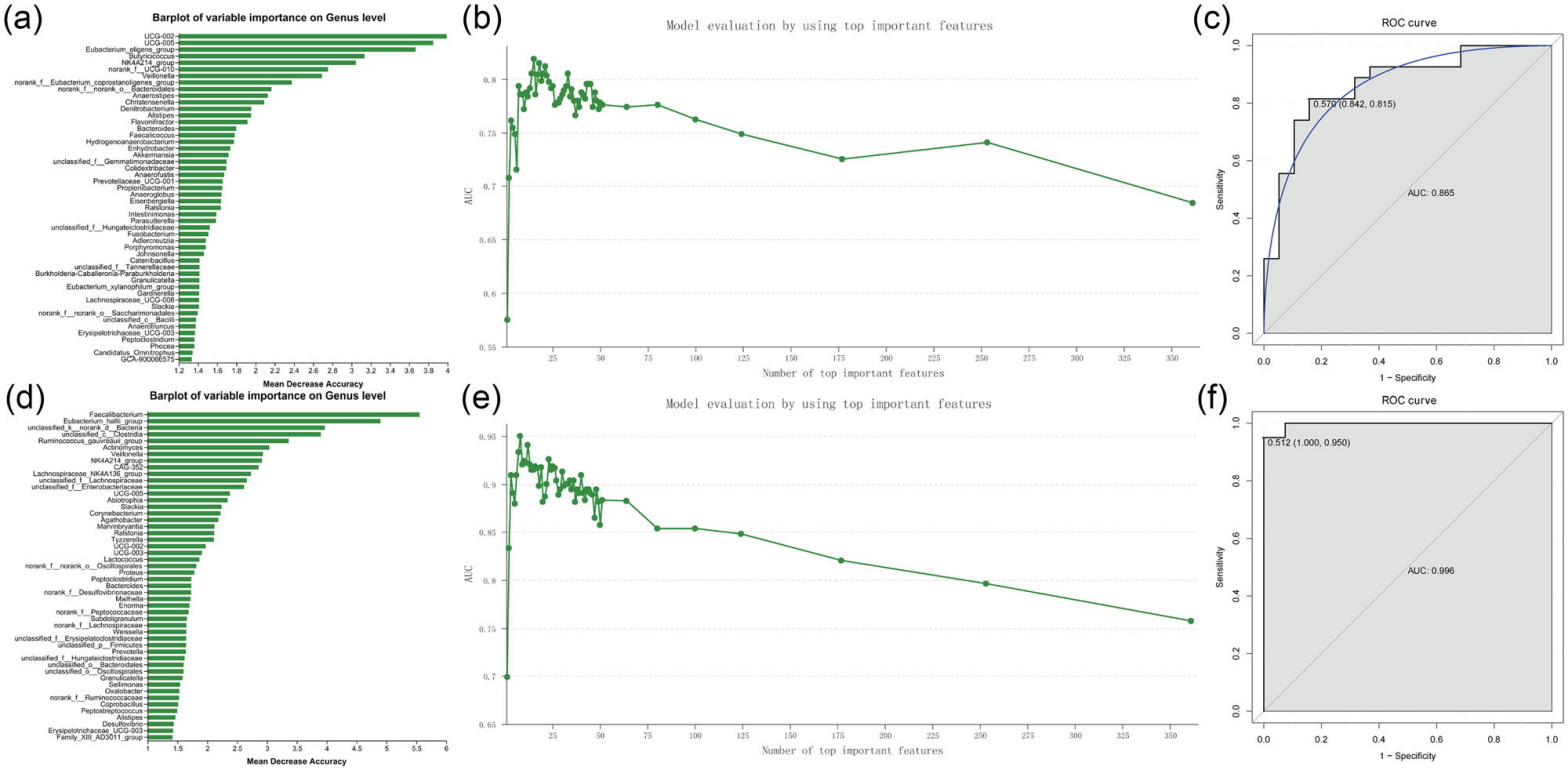
The sorting of gut microbiota species and the trend chart of AUC with increasing gut microbiota species in stroke (STR) and poststroke epilepsy (PSE): (a) the sorting of gut microbiota species in STR; (b) the trend chart of AUC with increasing gut microbiota species in STR; (c) the receiver operating characteristic (ROC) curve in STR; (d) the sorting of gut microbiota species in PSE; (e) the trend chart of AUC with increasing gut microbiota species in PSE; (f) the ROC curve in PSE.

A top‐down forward‐wrapper method was used to add one gut microbiota specie at a time (Figure 5a), and a total of 15 gut microbiota species were identified as the optimal diagnostic biomarkers for STR (Figure 5b), namely, *UCG‐002*, *UCG‐005*, *Eubacterium_eligens_group*, *Butyricicoccus*, *NK4A214_group*, *norank_f__UCG‐010*, *Veillonella*, *norank_f__Eubacterium_coprostanoligenes_group*, *norank_f__norank_o__Bacteroidales*, *Anaerostipes*, *Christensenella*, *Denitrobacterium*, *Alistipes*, *Flavonifractor*, and *Bacteroides*. The AUC value was 0.865 (Figure 5c), which indicated that the above gut microbiota species showed good diagnostic value for STR. In addition, a total of eight gut microbiota species were identified as the optimal diagnostic biomarkers for PSE (Figure 5d,e), namely, *Faecalibacterium*, *Eubacterium_hallii_group*, *unclassified_k__norank_d__Bacteria*, *unclassified_c__Clostridia*, *Ruminococcus_gauvreauii_group*, *Actinomyces*, *Veillonella*, and *NK4A214_group*. The AUC value was 0.996 (Figure 5f), indicating a good diagnostic value of the above gut microbiota species for PSE. It is noted that two common optimal diagnostic biomarkers for STR and PSE were identified, including *NK4A214_group* and *Veillonella*.

### 3.4 Functional prediction of gut microbiota species in STR and PSE

**FIGURE 6:**
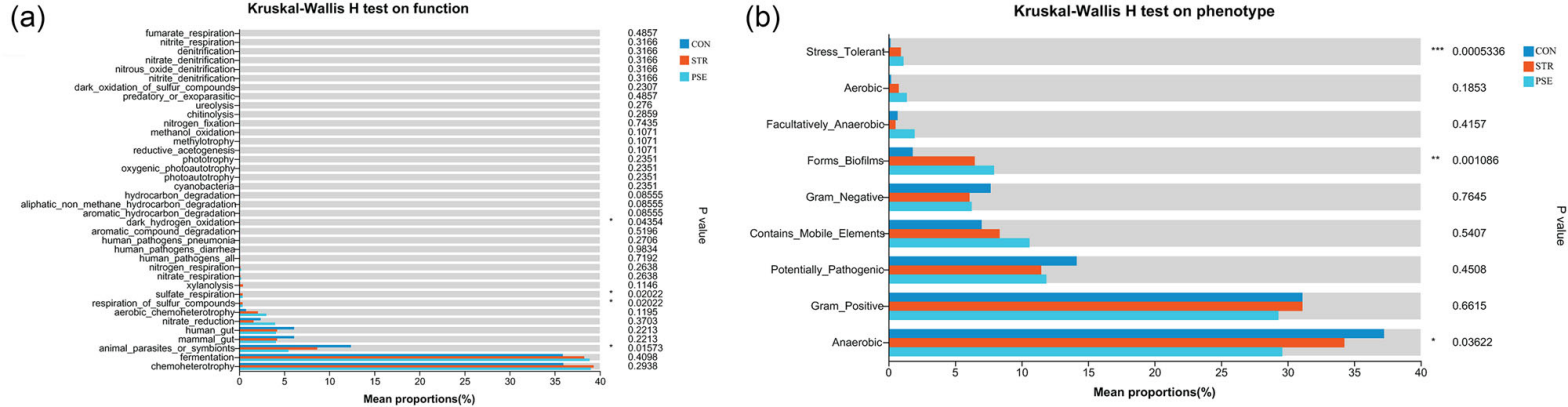
FAPROTAX and BugBase analysis of gut microbiota species in stroke (STR) and poststroke epilepsy (PSE): (a) FAPROTAX analysis; (b) BugBase analysis. ^*^.01 < *p* < .05; ^**^.001 < *p* < .01; ^***^.0001 < *p* < .001.

To explore the potential function of gut microbiota species in STR and PSE, the function prediction of FAPROTAX, BugBase, and PICRUSt2 (KEGG pathway, MetaCyc, and Module) was performed. In the FAPROTAX analysis, the functional component of respiration_of_sulfur_compounds and animal_parasites_or_symbionts was significantly increased and decreased in both STR and PSE, respectively (Figure 6a). In the BugBase analysis (Figure 6b), functional components of Stress_Tolerant and Forms_Biofilms were significantly increased in both STR and PSE. The functional component of anaerobic was significantly decreased in both STR and PSE.

**FIGURE 7:**
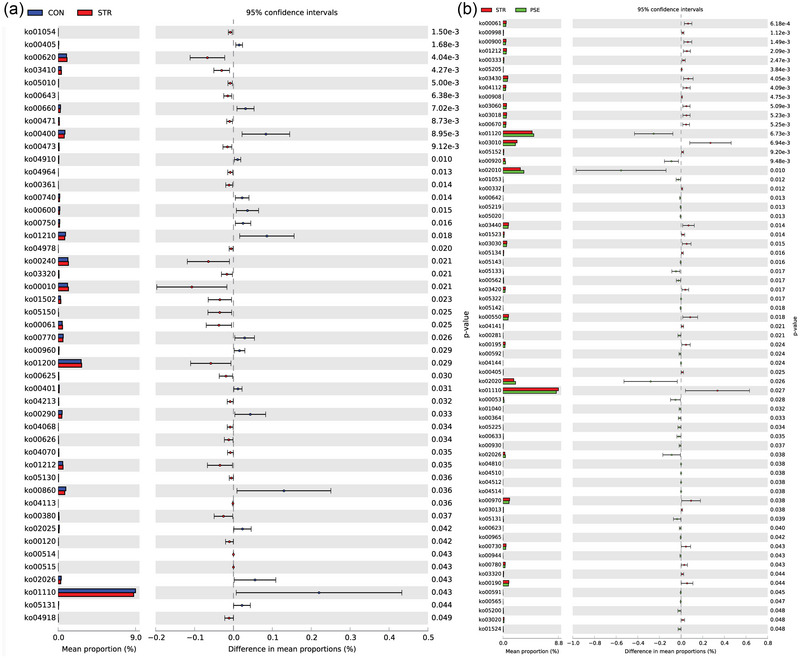
KEGG pathway analyses of gut microbiota species in stroke (STR) and poststroke epilepsy (PSE): (a) analysis in STR versus CON; (b) analysis in PSE versus STR.

**FIGURE 8:**
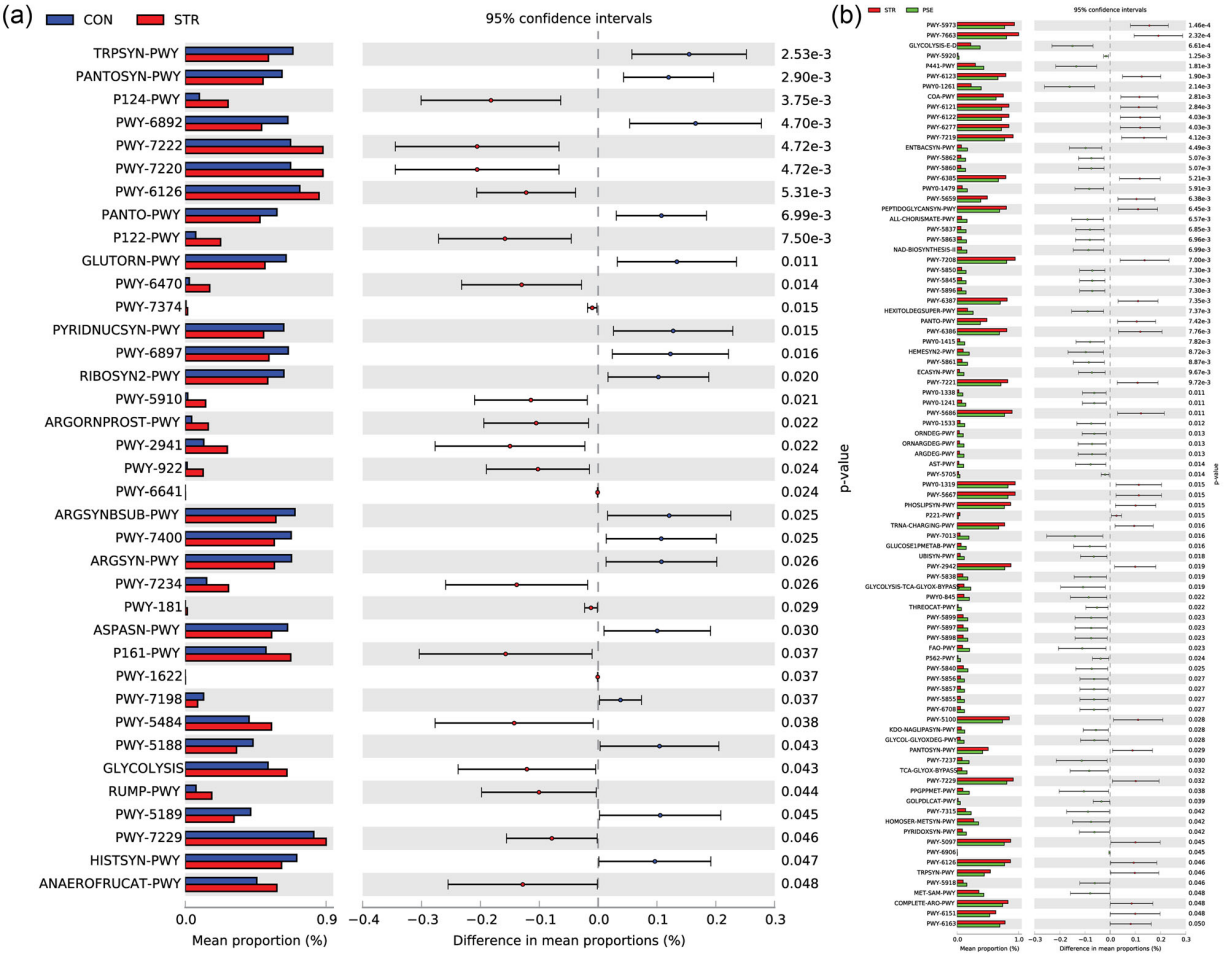
MetaCyc analyses of gut microbiota species in stroke (STR) and poststroke epilepsy (PSE): (a) analysis in STR versus CON; (b) analysis in PSE versus STR.

**FIGURE 9:**
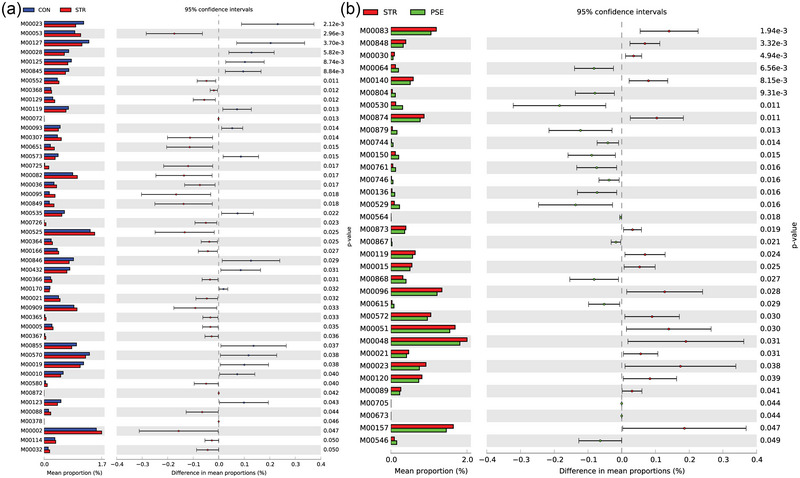
Module analyses of gut microbiota species in stroke (STR) and poststroke epilepsy (PSE): (a) analysis in STR versus CON; (b) analysis in PSE versus STR.

In the KEGG pathway analysis, ko01054 (nonribosomal peptide structures) and ko00405 (phenazine biosynthesis) were the most significantly changed functional component in STR compared with CON (Figure 7a). The ko01120 (microbial metabolism in diverse environments) and ko00061 (fatty acid biosynthesis) were the most significantly increased and decreased functional component in PSE compared with STR (Figure 7b). In the MetaCyc analysis, P124‐PWY (bifidobacterium shunt) and TRPSYN‐PWY (l‐tryptophan biosynthesis) were the most significantly changed functional component in STR compared with CON (Figure 8a). PWY‐5973 (cis‐vaccenate biosynthesis) was the most significantly decreased functional component in PSE compared with STR (Figure 8b). In the Module analysis, M00053 (pyrimidine deoxyribonucleotide biosynthesis) and M00023 (tryptophan biosynthesis) were the most remarkably changed functional component in STR compared with CON (Figure 9a). M00064 (ADP‐l‐glycero‐d‐manno‐heptose biosynthesis) and M00083 (fatty acid biosynthesis) were the most significantly changed functional component in PSE compared with STR (Figure 9b).

## 4 DISCUSSION

This was one of the first studies characterizing the gut microbiota in patients with PSE, filling the gut microbiota information gap in PSE. In STR and PSE, a significant dysbiosis of microbiome composition and function was present, implying the association between dysbiosis of microbial composition and function and STR and PSE. Additionally, our study gave new clues to explore the novel diagnostic biomarkers and interventions for PSE.

In the difference analysis of gut microbiota species in STR and PSE, *Enterococcus* was dominant gut microbiota species in STR at genus level. Moreover, the relative abundance of *butyricicoccus* was the most markedly reduced in STR compared with CON at the genus and genus levels. *Enterococcus* produces histamine, which functions as a neurotransmitter essential for modulating neuro‐inflammation in the brain (Alkasir et al., 2017). The relative abundance of *Enterococcus* is increased in patients with ischemic STR and cerebral hemorrhage (Haak et al., 2021; Tang et al., 2022). *Butyricicoccus*, the beneficial bacteria with anti‐inflammatory properties, can produce butyrate that helps maintain the integrity of the gut membrane (Canani et al., 2011; Hamer et al., 2008). In cerebral ischemic STR, supplementation rich in SCFA‐producing bacteria significantly increases the population of *Butyricicoccus*, thereby inhibit neuronal apoptosis and cerebral infarction volume and prevent neurobehavioral impairments (Chidambaram et al., 2022). It is indicated that *Enterococcus* and *butyricicoccus* may be associated with inflammation neurobehavior in the development of STR.

In addition, *E. Shigella* was dominant gut microbiota species in PSE at the genus level. Moreover, *C. innocuum‐group* and *Faecalibacterium* were the most significantly changed in PSE compared with STR. *Escherichia spp*. is capable of producing neurotransmitter gamma aminobutyric acid in host organism (Strandwitz & Kim, 2019). It is found that *E. Shigella* is significantly increased in infants with epilepsy compared with healthy infants (Liu et al., 2022). It is assumed that *E. Shigella* may be involved in the occurrence of epilepsy through inflammatory mechanisms and could be considered a potential marker to classify possible subtypes of epileptic diseases (Liu et al., 2022; Zhou et al., 2022). Members of the genus *Clostridium* form a crucial part of the anaerobic microflora and cause endogenous and exogenous infections. It is reported that *C. innocuum* induces edema, inflammation, and necrosis in a mouse ileal loop mode (Cherny et al., 2021). *C. innocuum* also participates in the regulatory process of gut microbiota on neurotransmitter synthesis and functions in the gut–brain axis (Chen et al., 2021). *Faecalibacterium*, beneficial microbes, can produce a high proportion of the SCFA butyrate in the intestine, which has anti‐inflammatory effects (Duncan et al., 2002; Qiu et al., 2013). It is found that the decrease of *Faecalibacterium* is associated with focal epilepsy (Zhou et al., 2022). It is assumed that *Faecalibacterium* could be involved in the mechanisms of epilepsy through the SCFAs pathways (Zhou et al., 2022). It is suggested that *E. Shigella*, *C. innocuum‐group*, and *Faecalibacterium* may be involved in the process of PSE through neurotransmitter synthesis and inflammatory mechanisms.

It is noted that the relative abundance of *Anaerostipes* was markedly decreased in both STR and PSE at the genus level and the species level. *Anaerostipes*, beneficial bacteria, translates propionate to butyrate, which has the ability to be anti‐inflammatory via inactivating the microglia (Yamawaki et al., 2018). The abundance of *Anaerostipes* is decreased in acute ischemic STR (Tan et al., 2021). It is found that the decrease of *Anaerostipes* is also associated with focal epilepsy (Zhou et al., 2022). Therefore, *Anaerostipes* may be involved in mechanisms of STR and PSE through the anti‐inflammatory mechanisms.

Based on diagnostic analysis in STR and PSE, a total of 15 gut microbiota species were identified as the optimal diagnostic biomarkers for STR, such as *Butyricicoccus*. In addition, a total of eight gut microbiota species were identified as the optimal diagnostic biomarkers for PSE, such as *Faecalibacterium*. It is worth mentioning that two common optimal diagnostic biomarkers for STR and PSE were identified, including *NK4A214_group* and *Veillonella*. The average relative abundance of *Ruminococcaceae NK4A214 group* is significantly higher in the spinal cord injury (Pang et al., 2022). Compared to low risk individuals, some high risk individuals with STR exhibited high levels of *Veillonellaceae* (Chidambaram et al., 2022). Increased abundance of *Veillonella* has been found in epilepsy with diarrhea patients (Liu et al., 2022). It is indicated that *NK4A214_group* and *Veillonella* are key pathogen in the disease development from STR to PSE and can be regarded as potential diagnostic markers for both STR and PSE patients.

According to the BugBase analysis of gut microbiota species, functional component of Stress_Tolerant was significantly increased in both STR and PSE. The functional component of anaerobic was significantly decreased in both STR and PSE. In vivo, STR can reduce blood flow and endothelial shear stress (Andjelkovic et al., 2020). The mechanism involved in ischemic STR all result in significant cerebral hypoperfusion, leading to an increase in anaerobic metabolism and causes an increase in neuroinflammatory cytokines (Peng et al., 2022). Due to epilepsy, increased neural activity leads to the accumulation of anaerobic glycolysis products in the neural tissue sand postictal enhancement of blood flow (Tatlidil, 2000). Thus, it can be seen that Stress_Tolerant and anaerobic play an important role in the development of STR and PSE.

In the KEGG pathway analysis, the fatty acid biosynthesis was the most markedly decreased functional component in PSE compared with STR. The therapeutic benefits of omega‐3 fatty acids for epilepsy have been expected due to the reduction of cardiac arrhythmia in omega‐3 polyunsaturated fatty acids (Calder & Yaqoob, 2010). It is indicated that fatty acid biosynthesis is associated with PSE. In the MetaCyc analysis, l‐tryptophan biosynthesis was the most significantly decreased functional component in STR compared with CON. Microbial compounds and metabolites from intestinal flora, including tryptophan, are important media for communication between the intestinal tract and the brain (Durgan et al., 2019). An electrophysiological study showed that elevated tryptophan concentration suppressed neuron firing (Gallager & Aghajanian, 1976). It is indicated that l‐tryptophan biosynthesis is associated with STR and may be involved in the process of PSE.

There were some limitations in this study. First of all, the sample size of this study was small. Therefore, it was necessary to expand the sample size for follow‐up research and further verify it in different populations. Second, this study lacked in vitro studies. Fortunately, in vitro experiments are currently being conducted, aimed at providing support for the conclusions of this study.

## 5 CONCLUSION

The disordered profiles of gut microbiota in STR and PSE were illustrated, such as the increase of *Enterococcus* and the decrease of *butyricicoccus* in STR, the increase of *E. Shigella* and *C. innocuum‐group* and the decrease of *Faecalibacterium* in PSE, and the decrease of *Anaerostipes* in both STR and PSE. Besides, potential diagnostic biomarkers for STR (*butyricicoccus*), PSE (*Faecalibacterium*), STR, and PSE (*NK4A214_group* and *Veillonella*) were also identified. Finally, some significantly dysfunctional components were identified, including l‐tryptophan biosynthesis in STR, fatty acid biosynthesis in PSE, and Stress_Tolerant and anaerobic in both STR and PSE. Our study might facilitate the diagnosis and treatment of PSE.

## AUTHOR CONTRIBUTIONS

**Duncan Wei**: Conceptualization; methodology; resources; data curation; formal analysis; writing—original draft; writing—review and editing. **Xiaopu Chen**: Conceptualization; methodology; data curation; formal analysis; writing—original draft; writing—review and editing. **Jing Xu**: Resources; data curation; formal analysis. **Xiaotang Peng**: Resources; data curation; formal analysis. **Shunxian Li**: Resources; data curation; formal analysis. **Yongling Yin**: Supervision; resources; data curation; formal analysis. **Wenzhen He**: Conceptualization; methodology; resources; data curation; formal analysis

## CONFLICT OF INTEREST STATEMENT

The authors declare that they have no conflicts of interest.

### CONSENT FOR PUBLICATION

The subjects gave written informed consent for the publication of any associated data.

### PEER REVIEW

The peer review history for this article is available at https://publons.com/publon/10.1002/brb3.3318.

## Data Availability

The datasets generated and analyzed during the current study are not publicly available but are available from the corresponding author on reasonable request.
